# Gut microbiota signature of pathogen-dependent dysbiosis in viral gastroenteritis

**DOI:** 10.1038/s41598-021-93345-y

**Published:** 2021-07-06

**Authors:** Taketoshi Mizutani, Samuel Yaw Aboagye, Aya Ishizaka, Theophillus Afum, Gloria Ivy Mensah, Adwoa Asante-Poku, Diana Asema Asandem, Prince Kofi Parbie, Christopher Zaab-Yen Abana, Dennis Kushitor, Evelyn Yayra Bonney, Motoi Adachi, Hiroki Hori, Koichi Ishikawa, Tetsuro Matano, Kiyosu Taniguchi, David Opare, Doris Arhin, Franklin Asiedu-Bekoe, William Kwabena Ampofo, Dorothy Yeboah-Manu, Kwadwo Ansah Koram, Abraham Kwabena Anang, Hiroshi Kiyono

**Affiliations:** 1 The Institute of Medical Science, The University of Tokyo, 4-6-1 Shirokanedai, Minato-ku, Tokyo, 108-8639 Japan; 2 Noguchi Memorial Institute for Medical Research, University of Ghana, Accra, Ghana; 3 Joint Research Center for Human Retrovirus Infection, Kumamoto University, Kumamoto, Japan; 4 AIDS Research Center, National Institute of Infectious Diseases, Tokyo, Japan; 5 Mie University, Mie, Japan; 6 National Mie Hospital, Mie, Japan; 7 Ghana Health Service, Accra, Ghana; 8 Institute for Global Prominent Research, Graduate School of Medicine, Chiba University, Chiba, Japan; 9 CU-UCSD Center for Mucosal Immunology, Allergy and Vaccines (cMAV), Department of Medicine, University of California San Diego, San Diego, CA USA

**Keywords:** Infectious diseases, Epidemiology

## Abstract

Acute gastroenteritis associated with diarrhea is considered a serious disease in Africa and South Asia. In this study, we examined the trends in the causative pathogens of diarrhea and the corresponding gut microbiota in Ghana using microbiome analysis performed on diarrheic stools via 16S rRNA sequencing. In total, 80 patients with diarrhea and 34 healthy adults as controls, from 2017 to 2018, were enrolled in the study. Among the patients with diarrhea, 39 were norovirus-positive and 18 were rotavirus-positive. The analysis of species richness (Chao1) was lower in patients with diarrhea than that in controls. Beta-diversity analysis revealed significant differences between the two groups. Several diarrhea-related pathogens (e.g., *Escherichia*-*Shigella*, *Klebsiella* and *Campylobacter*) were detected in patients with diarrhea. Furthermore, co-infection with these pathogens and enteroviruses (e.g., norovirus and rotavirus) was observed in several cases. Levels of both Erysipelotrichaceae and Staphylococcaceae family markedly differed between norovirus-positive and -negative diarrheic stools, and the 10 predicted metabolic pathways, including the carbohydrate metabolism pathway, showed significant differences between rotavirus-positive patients with diarrhea and controls. This comparative study of diarrheal pathogens in Ghana revealed specific trends in the gut microbiota signature associated with diarrhea and that pathogen-dependent dysbiosis occurred in viral gastroenteritis.

## Introduction

Diarrheal disease is a leading cause of mortality in young children in developing countries. Globally, 1.6 million people died from diarrhea in 2017^1^. Diarrheal morbidity and mortality in South Asia and sub-Saharan Africa are reported to be high, with one-third of the victims of diarrhea being under the age of five^2^. Viral (e.g., norovirus and rotavirus) and bacterial pathogens (e.g., *Escherichia*, *Shigella*, *Campylobacter and Vibrio cholerae*) are the primary causative agents of diarrheal diseases worldwide. Among them, rotavirus is one of the leading causes of fatal diarrhea disease in children^3^, which also infects adults, but with a reduced clinical symptom compared to that in children. Norovirus is also the most common cause of gastroenteritis worldwide in patients of all ages^4^. The prevalence of norovirus infection in Africa is unclear; however, several studies have reported the detection of norovirus by RT-PCR in stool samples from patients with diarrhea in African countries^5–7^.

*Vibrio*, *Shigella*, and *Campylobacter* are the major groups of organisms responsible for diarrheal diseases^8^. From a public health perspective, characterizing the causative pathogen of diarrhea that is predominant in a country can lead to timely preventive measures and the development of educational provisions for citizens to effectively counter the disease. To acquire fundamental information on the public health status of Africans, we analyzed the intestinal microenvironment in the Ghanaian population and reported the fecal microbiome composition in healthy Ghanaian adults^9^. In Ghana, similar to other African countries, diarrheal diseases occur throughout the year, giving rise to serious public health concerns^10^. Several cholera outbreaks have occurred in Ghana, including in 2014, when 28,975 cholera cases with 243 deaths were reported in all 10 regions of the country^11^. Cholera outbreaks occur sporadically every year in Ghana and unsanitary living conditions influence the occurrence of such outbreaks; therefore, it is crucial to improve these conditions. Poor sanitary conditions heighten the risk of developing diarrheal diseases in community settings and issues related to sewage treatment significantly influence the prevalence of diarrhea. In fact, temporary spells of heavy rains, such as squalls, that frequently occur in tropical areas tend to greatly impact the sanitary environment. Most diarrheal pathogens are transmitted through contaminated food, water, or the fecal–oral route. Several studies have shown that certain causative pathogens of diarrhea are often isolated from street food fare prepared in huts^12,13^. However, studies focusing on multiple etiologies are inadequate, and there is limited research focusing on the causal pathogens of diarrheal disease in developing countries^14^.

In Ghana, the diagnoses of diarrheal diseases in hospitals and reference laboratories are mostly based on the rapid diagnosis of Vibrio Cholera. Therefore, it is important to understand the prevalence and pathogens of diarrheal diseases in order to facilitate accurate medical practice. In the present study, we examined the profile of gut microbiota during diarrheal episodes caused by viral or non-viral etiological agents in patients (adults, adolescent and children) in Ghana. We further evaluated the differences in the gut microbiota communities between enterovirus-positive and enterovirus-negative diarrheic stool samples.

## Results

### Cohort clinical characteristics

Overall, 80 diarrheic stool samples, during 2017–2018, from patients comprising of 49 adults (> 19 years old), 13 adolescents (10–19 years old), and 18 children (< 10 years old) were analyzed. Forty-eight (60.0%) patients were female. There were a total of 39 norovirus-positive patients and 18 rotavirus-positive patients (Table 1). Thirty-four healthy adult individuals were enrolled in the present study as controls.

**Table 1:** Clinical and demographic characteristics.

Description	Category	Cases (n = 80)	Healthy (n=34)	p-value^1^
.Age: n, median, (IQR^2^)	Child (0–10 years)	18, 3 (1–6)	0	ND
	Adolescent (11–19 years)	13, 15 (13–17)	0	ND
	Adult (> 19 years)	49, 33 (25–45)	34, 45 (31–50)	0.065
Gender: n (ratio %)	Male	32 (40.0%)	9 (26.5%)	ND
	Female	48 (60.0%)	25 (73.5%)	ND
Viral detection: n (ratio %)	None	23 (27.0%)	ND	ND
	Norovirus	39 (45.9%)	ND	ND
	Rotaviruis	18 (21.1%)	ND	ND
Antibiotics: n (ratio %)	Present	17 (21.3%)	0 (0%)	ND
	Absent	50 (62.5%)	34 (100%)	ND
	Unknown	13 (16.2%)	0 (0%)	ND

^1^Statistical comparison between healthy controls and diarrhea adult patients was performed by Wilcoxon rank sum test or Fisher's exact test.

^2^Interquartile range.

^3^Not determined.

### Fecal microbiome composition in Ghanaian patients with diarrhea

**Figure 1:**
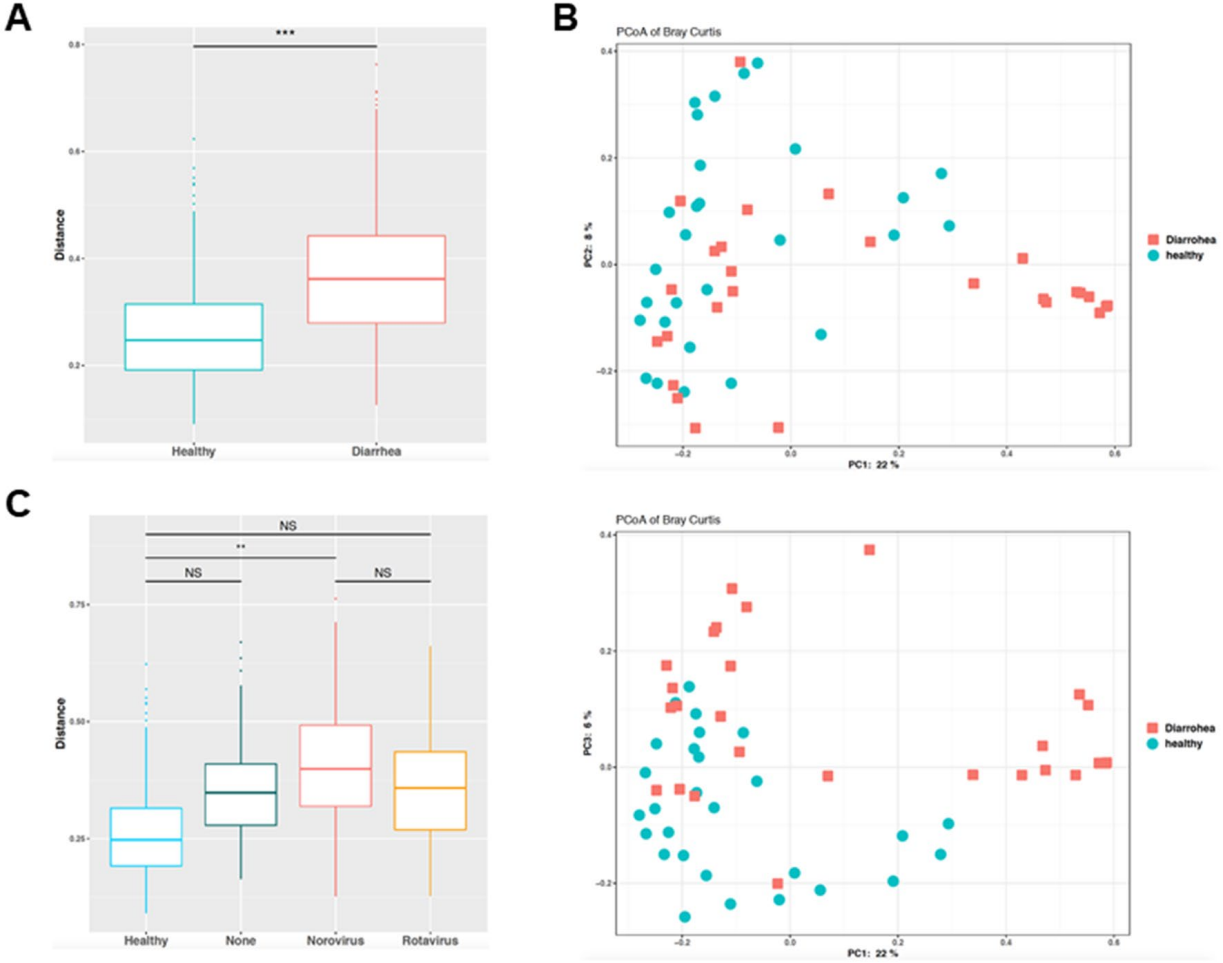
Beta diversities in fecal microbiome in adult patients with diarrhea and healthy adult cohorts. (**A**) Weighted Unifrac distances of fecal microbiome in individuals with diarrhea and healthy adult cohorts. Weighted unifrac distances to healthy controls indicates that individuals with diarrhea possess significantly distant fecal microbiome composition (PERMANOVA, q = 0.002). (**B**) Principal coordinate analysis (PCoA) plots based on Bray–Curtis diversity metric. (**C**) Comparing weighted Unifrac distances of fecal microbiome among individuals with diarrhea including viral none-detectable diarrhea (None), norovirus detected diarrhea (Norovirus), and rotavirus detected diarrhea (Rotavirus), to healthy controls. Fecal microbiome composition of individuals with Norovirus infection are significantly distant from healthy controls (PERMANOVA, q = 0.01). **, *** indicate PERMANOVA significant differences with q =  < 0.01, and < 0.005 respectively.

Although diarrhea is not always caused by infection with pathogens, we hypothesized the presence of pathogens and sequenced the V3–V4 hypervariable region of the 16S rRNA gene in 80 stool samples. First, we compared and analyzed the fecal bacterial profiles of diarrhea in adult patients and healthy adults. We then determined the alpha-diversity of the stool samples by calculating the Shannon index and Chao1 index in QIIME2. The analyses were performed with samples rarefied to a read depth of 10,000 to ensure that a reasonable number of sequence reads were obtained for each operational taxonomic unit (Supplemental Figure 1A). There was no difference in species evenness according to the Shannon index between both groups Supplemental Figure 1B, q = 0.11), whereas species richness according to the Chao1 index demonstrated significant differences between both groups (Supplemental Figure 1C, q = 0.002). Furthermore, beta-diversity in the fecal microbiome was assessed by the weighted Unifrac algorithm. Gut bacterial beta-diversity was significantly different between the two groups (Fig. 1A, q = 0.002). Using these matrices for permutational analysis of variance (PERMANOVA) and principal coordinates analysis (PCoA), variation in microbiome community structure was observed between both groups (Fig. 1B). To explore the viral pathogen-specific dysbiosis, we next investigated the bacterial community in each gut microenvironment in norovirus- or rotavirus-infected adult patients with diarrhea. We separated patients with diarrhea into three groups (viral non-detectable, norovirus infection, and rotavirus infection) based on the results of the viral test and then performed beta-diversity analysis at the genus level to examine their fecal microbiome (Fig. 1C). This analysis showed that each distance of the bacterial composition between above three groups in the patients with diarrhea was not significant in pairwise permutational analysis of variance. The differences in the bacterial composition between patients with diarrhea caused by norovirus infection and healthy controls was significant (q = 0.01); however, the differences between the healthy control and rotavirus-positive diarrhea, and between the healthy control and viral-negative diarrhea, were not significant, respectively (q = 0.05, q = 0.087). No statistical difference in the bacterial composition was found between norovirus-positive and rotavirus-positive diarrhea (q = 0.88, Fig. 1C).

### Gut bacteriome profiles in diarrheal patients and healthy individuals

**Figure 2:**
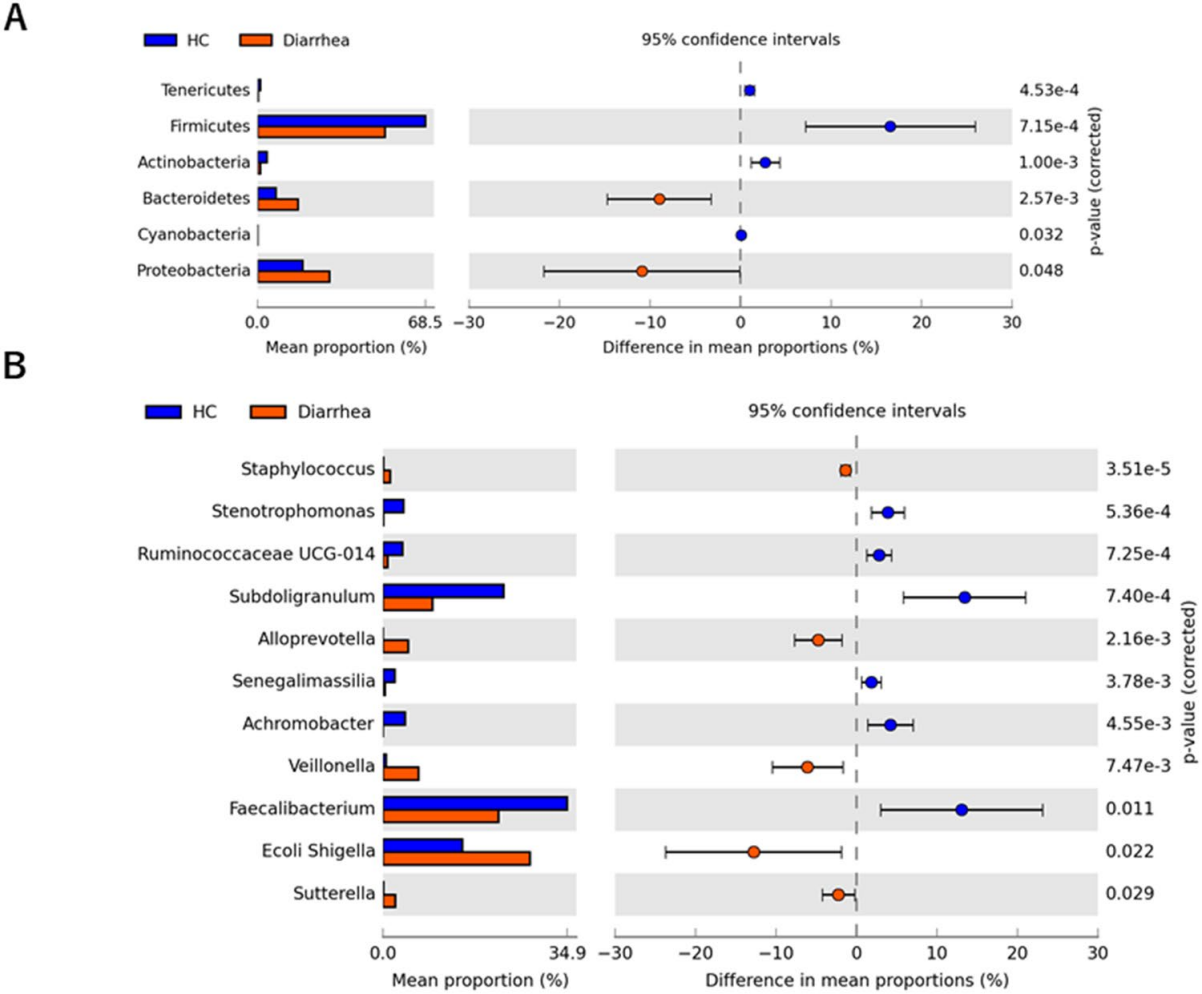
Fecal bacteria abundance at the phylum level by STAMP analysis. Comparison of gut microbiota between the healthy adult controls and adult patients with diarrhea by STAMP analysis at the phylum level (**A**) and genus level (**B**). *HC* healthy adult control, *Diarrhea* adult patient with diarrhea.

**Figure 3:**
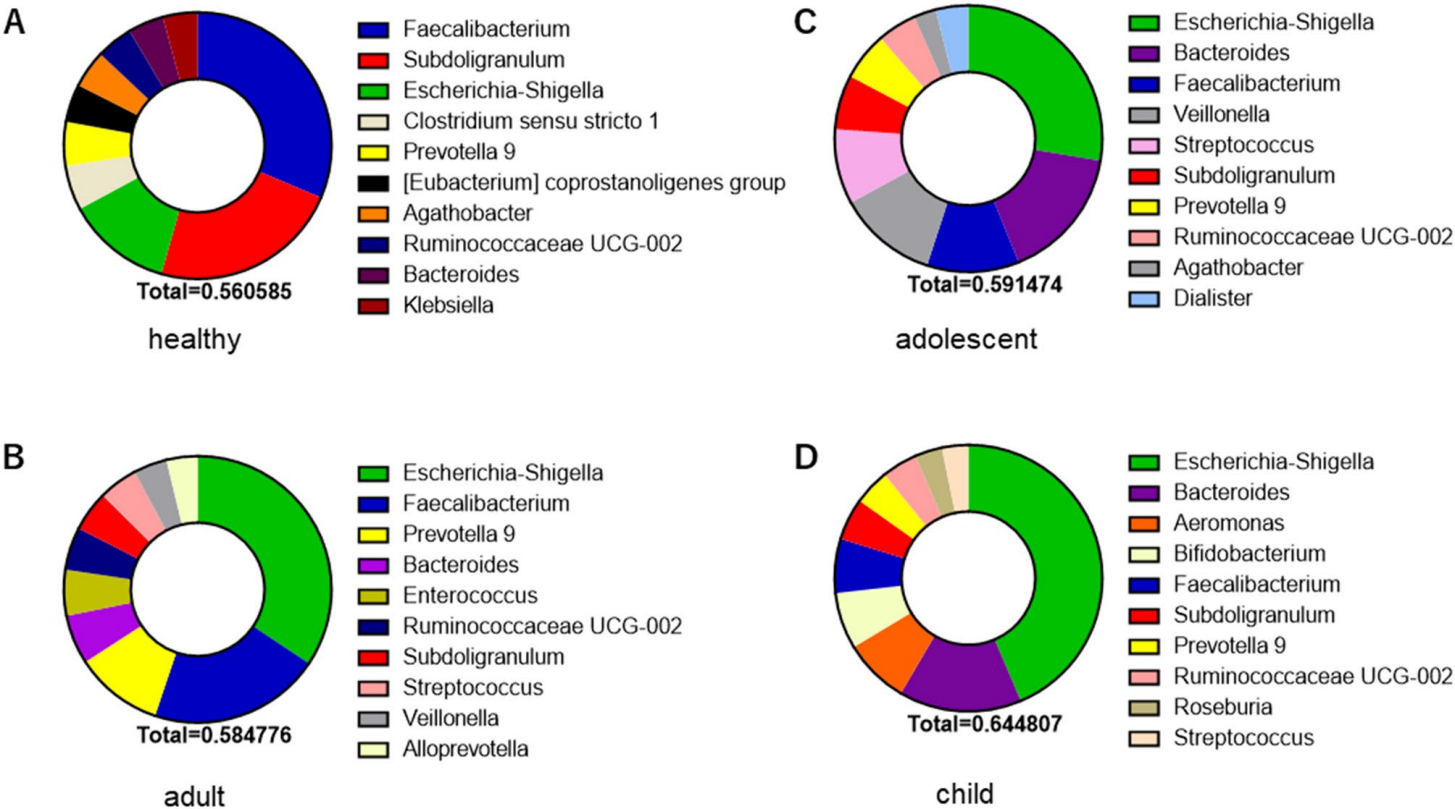
Taxonomic profiles (genus) of fecal bacteria in patients with diarrhea and healthy controls. Top 10 relative abundance of fecal taxa at the phylum level in healthy adult controls (**A**) and patients with diarrhea; adults (**B**), adolescents (**C**), and children (**D**).

The top 10 phyla showing the highest relative abundance of fecal bacteria in healthy Ghanaian adults and patients with diarrhea are shown in Supplemental Figure 2A and 2B. *Firmicutes*, *Proteobacteria*, and *Bacteroidetes* were the predominant phyla in both healthy adults and adult diarrheal patients, although the ratios of the mean relative abundance differed. A similar profile was observed in adolescent patients with diarrhea (Supplemental Figure 2C), whereas *Proteobacteria* was the most dominant phylum in children with diarrhea (Supplemental Figure 2D). STAMP analysis^15^ revealed that Tenericutes, Firmicutes, Actinobacteria, Bacteroides, Cyanobacteria, and Proteobacteria statistically differed between healthy adults and adult diarrheal patients (Fig. 2A). Next, the top 10 genera showing the highest relative abundance of fecal bacteria in each category of patients with diarrhea (adult, adolescent, and child) and healthy adults are shown in Fig. 3. *Faecalibacterium* (17.6%), *Subdoligranulum* (12.9%), and *Escherichia-Shigella* (7.1%) were the dominant genera in healthy adults (Fig. 3A)*.* However, *Escherichia-Shigella* were the most abundant genera among all ages in patients with diarrhea (adult, 20%; adolescent, 16.4%; child, 28%) (Fig. 3B–D).

### Gut bacterial composition in patients with diarrhea

**Figure 4:**
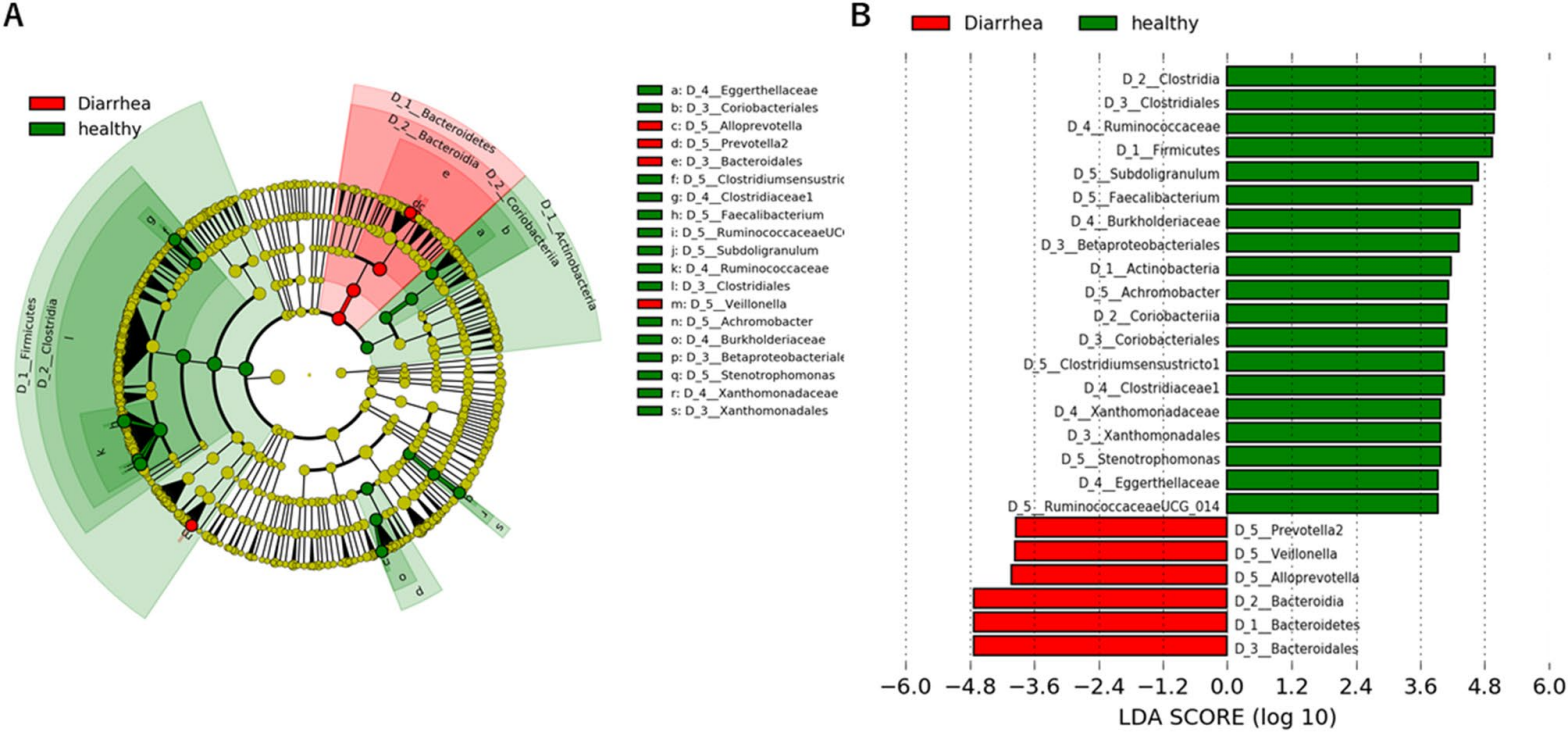
Comparison of fecal microbiota between adult patients with diarrhea and healthy controls. Identification of difference of bacterial markers in gut microbiota between adult patients with diarrhea and healthy controls using LEfSe analysis (**A**) and LDA score > 3.0 (**B**). The cladogram was calculated and depicted by LEfSe, a major metagenomic analysis method. The effect size of each taxon or OUT with different quantities was evaluated by Wilcoxon sum-rank test followed by liner discriminant analysis.

In STAMP analysis at genus level, compared with healthy subjects, the relative abundances of the genera *Staphylococcus*, *Veillonella, Alloprevotella, Escherichia-Shigella*, and *Sutterella* were significantly elevated in adult patients with diarrhea (Fig. 2B). In contrast, *Faecalibacterium*, *Stenotrophomonas*, and *Subdoligranulum*, which are dominant genera in healthy Ghanaian adults, were significantly decreased in adult patients with diarrhea. To examine the specific bacterial taxa associated with diarrhea, we compared the bacterial composition in healthy adults and patients with diarrhea using linear discriminate analysis effect size (LEfSe)^16^. A cladogram shows the structure of the fecal bacteria and the predominant bacteria in healthy adults and patients with diarrhea. At the phylum levels, Firmicutes, Actinobacteria and Bacteroidetes were mainly altered in patients with diarrhea in comparison with the profiles of the corresponding phyla in healthy controls (Fig. 4A). Changes in the composition of gut microbiota between healthy adults and patients with diarrhea were analyzed at different taxonomic levels. LEfSe analysis identified 25 discriminative biomarkers (Linear discriminant analysis; LDA score > 3, Fig. 4B).

### Analysis for trends of causative pathogen in diarrheal patients

We further examined the ratio of bacterial sequence known as a causative pathogen of diarrhea in diarrheal stool samples. *Escherichia-Shigella* was the most abundant genus in diarrheal stool samples, although *Escherichia-Shigella* is also among the top 3 genera in healthy Ghanaian adults (Fig. 3 and Table 2). *Staphylococcus* and *Salmonella*, which are opportunistic bacteria, were interestingly detected more frequently in patients with diarrhea than in healthy subjects; additionally, *Vibrio* and *Campylobacter* were detected at a high ratio in all age groups (20%) whilst *Aeromonas* was mainly detected in the stool samples of children (12.6%). It is noteworthy that diarrheal stools with norovirus or rotavirus were found to have more bacteria at the genus level containing specific diarrheal causative bacteria than those of healthy subjects (Supplemental Figure 3), suggesting the possibility of co‐infection with virus and bacteria occurred in some diarrhea cases.

**Table 2:** Positive rate of fecal microbiome which is causative pathogen diarrhea.

Phylum	Class	Order	Family	Genus	Positive rate (%)	
					Healthy	Diarrhea
Proteobacteria	Gamma proteobacteria	Enterobacterales	Enterobacteriaceae	*Escherichia-Shigella*	98.1	98.9
				*Klebsiella*	91.0	71.0
				*Salmonella*	0.6	10.5
		Aeromonadales	Aeromonaceae	*Aeromonas*	0.1	12.6
		Vibrionales	Vibrionaceae	*Vibrio*	0.6	11.5
		Enterobacterales	Enterobacteriaceae	*Citrobacter*	0.1	4.2
Firmicutes	Bacilli	Bacillales	Staphylococcaceae	*Staphylococcus*	54.9	94.7
Proteobacteria	Epsilonproteobacteria	Campylobacterales	Campylobacteraceae	*Campylobacter*	4.9	22.1

### Inferred functional analysis with KEGG pathway in different causative diarrheal pathogen

**Figure 5:**
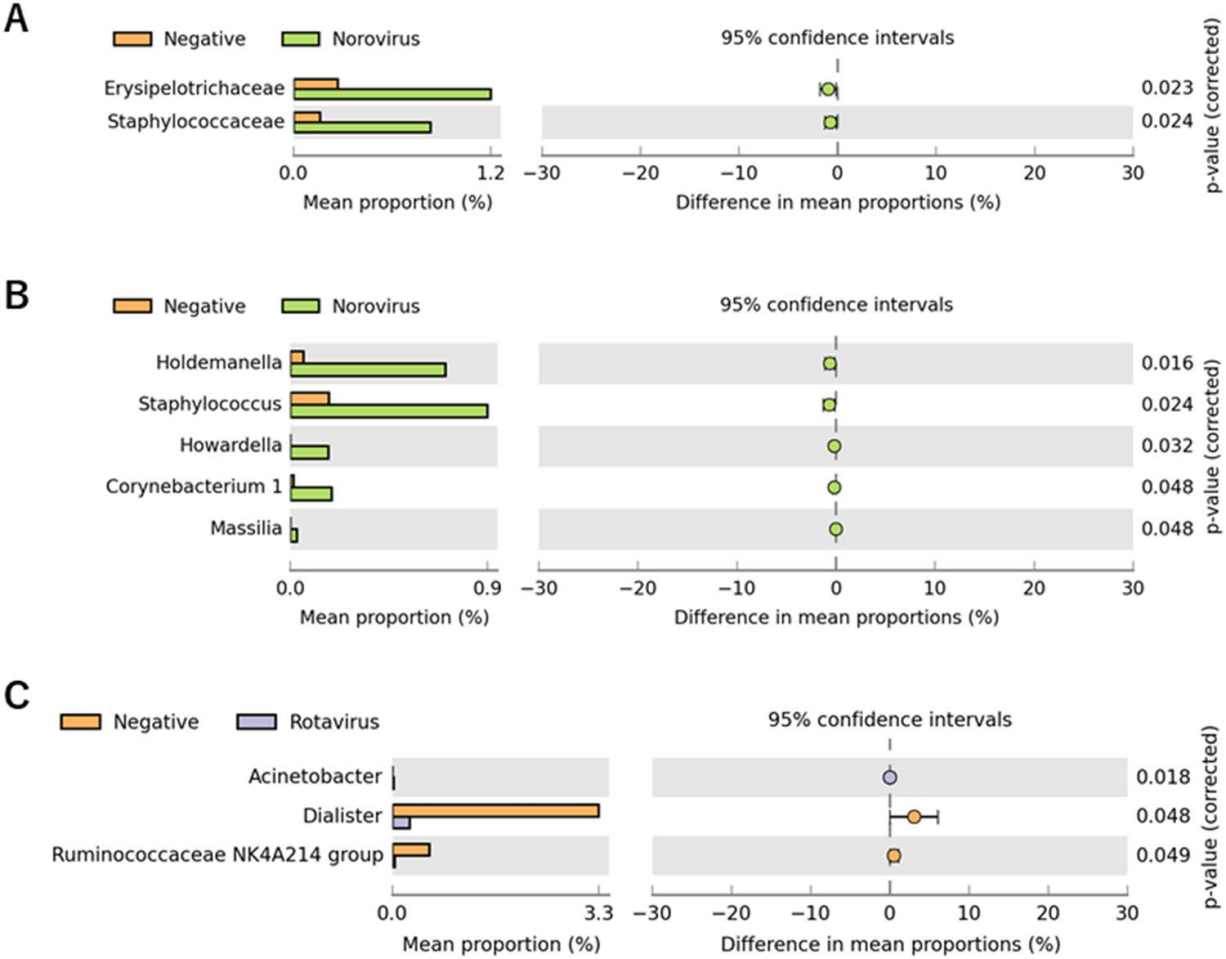
Comparing fecal bacteria abundance between enterovirus positive and negative diarrhea patients. Comparison of gut microbiota between enterovirus positive or negative diarrhea patients by STAMP analysis. (**A**) The difference of gut microbiota between norovirus detected and non-viral detected diarrhea patients at the family level (**A**) and genus level (**B**). The difference of gut microbiota between rotavirus positive and non-viral detected diarrhea patients at the genus level (**C**).

To explore the viral pathogens specific dysbiosis, we next, compared the community structure of microbiota among non-viral–detected and virus-detected diarrheal stool samples. At the family level, the proportions of Erysipelotrichaceae and Staphylococcaceae were markedly increased in norovirus positive diarrhea compared to the corresponding proportions in non-viral–detected diarrhea (Fig. 5A). At the genus level, *Holdemanella*, *Staphylococcus*, *Howardella*, *Corynebacterium 1*, and *Massilia* were significantly increased in stools in which norovirus was detected (Fig. 5B). In contrast, at the genus level, *Acinetobacter* was increased, whereas *Dialister* and *Ruminococcaceae NK4A214* group were decreased in rotavirus-infected stool samples (Fig. 5C). However, there was no significant difference at the family level in rotavirus-infected stool samples compared to non-viral–detected stools (data not shown).

**Figure 6:**
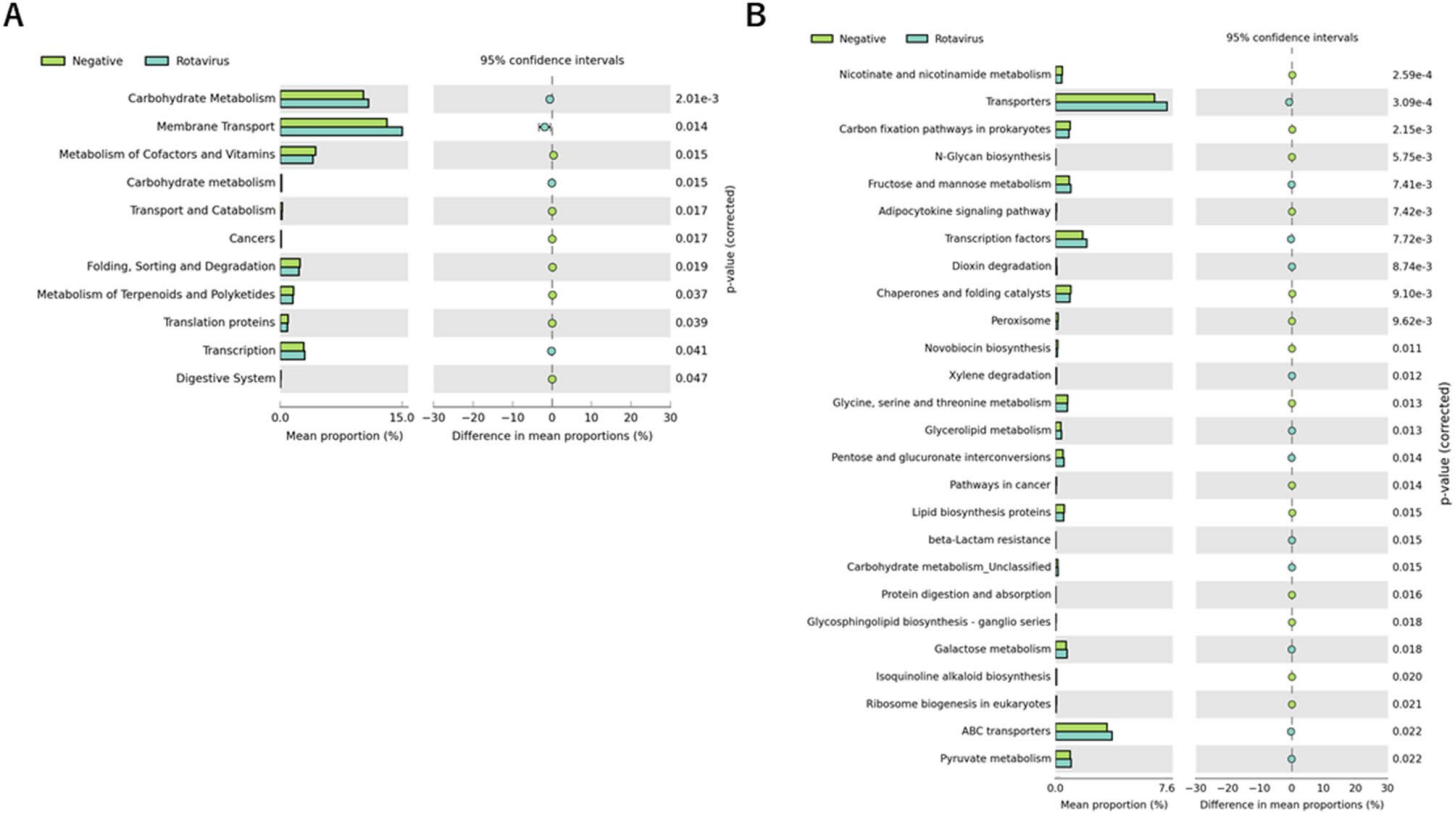
Distribution of Kyoto Encyclopedia of Genes and Genomes (KEGG) functional categories. Comparison between healthy controls and rotavirus-positive patients with diarrhea on levels 2 (**A**) and 3 (**B**) of KEGG. Functional contributions of the gut microbiota were analyzed using PICRUSt software.

Finally, we examined the predicted metabolic pathways and functions of the microbiota between non-viral–detected stool and virus-detected stool. A total of ten pathways at KEGG level 2 were significantly different between rotavirus-detected stool and non-virus-detected stool (Fig. 6A). We found the carbohydrate metabolism pathway to be different among the ten pathways (p = 0.002), although no differentially abundant KEGG pathway was found between norovirus-detected stool and non-virus–detected stool. Furthermore, KEGG level 3 analyses showed that changes in the carbohydrate metabolism pathway in rotavirus-positive stool were ascribed to alterations in fructose and mannose metabolism, pentose and glucuronate interconversions, galactose metabolism, and pyruvate metabolism (Fig. 6B).

## Discussion

Diarrheal morbidity and mortality rates in South Asia and sub-Saharan Africa are reported to be high^1^. However, although the causes of diarrheal pathogens in diarrhea-endemic countries are unknown, the main cause is thought to be a combination of unsanitary water, foods, and a poor hygienic environment. Knowledge of the trends modulating diarrheal pathogens would enable physicians to identify the causative agent and prescribe an appropriate treatment course for patients, consequently improving public health. Using NGS technology to analyze diarrheal pathogens in developing countries may lead to the discovery of unknown pathogens, which would be significant from the viewpoint of preventive medicine. In Ghana, diarrhea is a frequent infectious disease and an issue that requires urgent attention from a public health perspective^17^. In this study, we attempted to investigate trends in diarrheal diseases in Ghana using NGS analysis to advance our bench analyses of gut microbiota for the strengthening public health and surveillance systems for diarrheal diseases.

In Ghana, the diagnosis of diarrheal disease in hospitals and public health laboratories is mostly based on a rapid diagnosis for *Vibrio cholera*. Therefore, it was considered important to understand the prevalence and actual pathogenesis of diarrheal diseases to promote accurate medical practice. Our Science and Technology Research Partnership for Sustainable Development (SATREPS, AMED-JICA, Japan) project team selected a model area (Ga West Municipal Hospital, Greater Accra Region) and collaborated with local hospitals and research laboratories (NMIMR) for identification of the pathogens from diarrheal stool samples, using biochemical tests and NGS technology.

In our study, at the genus level, we detected *Campylobacter* more frequently in patients with diarrhea than in healthy controls (Supplemental Figure 3). *Campylobacter* is a pathogen that causes diarrhea worldwide and results in a high rate of childhood mortality in low- to middle-income countries^18,19^. Additionally, *Campylobacter* causes diarrhea, with an associated high rate of mortality, in breastfed infants in sub-Saharan Africa and South Asia^20^. A recent study reported that certain domestic animals in Ghana harbor multidrug-resistant *Campylobacter* species^21^. Therefore, there is a possibility of zoonotic transmission of *Campylobacter* species from animals to humans via direct contact or by the consumption of contaminated meat. Thus, from the standpoint of public health in Ghana, it is vital to continue monitoring the prevalence of *Campylobacter* and the rate of drug resistance. In addition, there is a need to build capacity within the public health institutes in Ghana for the inclusion of *Campylobacter* in routine diagnosis.

STAMP analysis showed that at the family level, the proportions of Erysipelotrichaceae and Staphylococcaceae were markedly increased in norovirus-induced diarrhea compared to the corresponding proportions in non-virus-induced diarrhea (Fig. 5A). Notably, a positive correlation was previously reported between Erysipelotrichaceae and inflammation^22^. One study reported that the abundance of Erysipelotrichaceae was increased in colorectal cancer^23^. Another study showed that the Erysipelotrichi class was positively correlated with tumor necrosis factor alpha in chronic HIV patients being administered suppressive anti-retroviral therapy^24^. Indeed, our unpublished observation is that genus *Erysipelotrichaceae UCG 003* were detected to be significantly abundant in Ghanaian HIV patients than that in controls. These reports indicate that immune system activation is associated with the symptoms of norovirus infection.

Collectively, the increased prevalence of Erysipelotrichaceae in patients with norovirus-induced diarrhea implies that it may be associated with inflammation in the intestinal tract. Conversely, at the genus level, *Dialister* and *Ruminococcaceae NK4A214* group were significantly decreased in patients with rotavirus-induced diarrhea compared to patients with non-virus-induced diarrhea (Fig. 5C). The reasons for the decline in these genera are unclear currently; however, a prior study reported that individuals with a higher proportion of *Ruminococcaceae* are less susceptible to rotavirus infection^25^. In a recent study, rotavirus-infected rats fed oligosaccharides showed improvement in diarrheal symptoms and rotavirus-associated dysbiosis; additionally, an increase in the proportion of *Ruminococcaceae* was observed^26^. Overall, a decrease in the *Ruminococcaceae* proportion due to rotavirus infection may negatively impact the intestinal environment of patients with diarrhea.

Gut dysbiosis due to viral infection probably includes the disproportionate expansion of potentially harmful bacterial species, reduction in the populations of common and beneficial bacterial species, and the resulting loss of diversity^27^. The differences in the gut microbiota between rotavirus and norovirus infections observed in our study are noteworthy (Fig. 5). This may be due to variations in crosstalk via direct interaction between rotaviruses and bacteria, as well as between noroviruses and bacteria, in the gut^28^. For example, almost all human norovirus strains were shown to bind to 'histo-blood group antigens' (HBGAs), which are blood type-associated glycoproteins expressed in numerous tissues in the body, including the intestinal tract. HBGAs are thought to be a supporting factor for norovirus infection and transmission in the host^28^. *Enterobacteriaceae* have been reported to harbor HBGA-like carbohydrates on their surfaces and have recently been shown to bind to noroviruses^29^.

In addition, norovirus has been reported to bind to sialic acid residues expressed on the bacterial surface^30^. Differences in the cytotoxicity of both viruses to the intestinal tract may also affect the output of dysbiosis of gut commensal microbiota^31,32^. Furthermore, bacteriophages are known to have a strong influence on bacterial diversity and population structure by infecting bacteria^33^. Additionally, these phages might affect dysbiosis in diarrhea, although the relation between enterovirus infection and phages is not known. Taken together, it is possible that these direct or indirect factors may have an impact on the gut environment.

The combination of microorganisms, such as viruses and the gut microbiota, probably plays an important role in shaping a healthy gut environment. Remarkably, co-infection with virus and bacterial groups containing pathogenic bacteria was highly prevalent in certain cases of diarrhea (Supplemental Figure 3), and this has been reported previously as well. One study from Africa showed that approximately 60% children under 5 years of age visiting the hospital due to episodes of acute diarrhea were infected with bacterial or viral pathogens, and 10% of them exhibited co-infections^34^. Additionally, studies from China and India reported that virus–bacteria or virus–parasite co-infection were detected in children with diarrhea under 5 years of age^35,36^. Several studies have discussed the implications of multiple pathogen-induced infection causing more severe diarrhea than infection with a single pathogen. For example, earlier studies reported that the combination of rotavirus and *Escherichia coli* co-infection intensifies the severity of diarrhea^3^. These co-infecting pathogens may also act synergistically, leading to even more serious illnesses and play a critical role in the incidence of severe gastroenteritis with diarrhea.

In this study, at genus levels, *Escherichia-Shigella* have been detected in most healthy Ghanaians. Since a certain species of *Escherichia-Shigella* is known to have pathogenic effect against humans, it is possible that pathogenic bacteria establish commensal relationship with the host. It is unclear how the pathogenic bacteria comprise commensal bacteria; however, the synergistic effect of new pathogens from outside and these commensal pathogenic bacteria may contribute to the severity of diarrhea. Although the mechanism has not been elucidated, our recent human clinical study of rice-based oral cholera vaccine (MucoRice-CTB) showed that high responders of MucoRice-CTB possessed increased *Escherichia-Shigella* in healthy Japanese volunteers^37^. We hypothesize that symbiosis of pathogens may lead to an enhanced intestinal immune response.

Overall, a thorough understanding of the biology of these pathogens and the interactions among co-infection pathogens is essential to decipher the pathogenesis of diarrheal diseases. Distinguishing between single and mixed infections may provide a detailed understanding of the pathogenesis of intestinal infections.

Our PICRUSt result speculated enrichment of the carbohydrate metabolism pathway in rotavirus-induced diarrhea (Figs. 5 and 6). Short-chain fatty acids are the major end products of carbohydrate digestion by bacteria and exert multiple effects on human health, including improvement of intestinal peristalsis. Rotaviruses infect enterocytes of the villi of the small intestine and replicate mainly in the gut^38^. Rotavirus-damaged enterocytes cannot absorb nutrients, and carbohydrates within food are metabolized by gut bacteria inhabiting the large intestine to produce higher-than-normal levels of short-chain fatty acids. Therefore, rotavirus-induced dysbiosis may accelerate intestinal transit, worsening the symptoms of patients with diarrhea, which may partially explain the occurrence of severe watery diarrhea in rotavirus infection. Further studies are warranted to gain insights into dysbiosis observed in patients with diarrhea.

This study is the first comparative analysis of different diarrheal pathogens and their associated intestinal microorganisms in the Ghanaian population. Notably, our data revealed that several bacterial taxa with potential pathogenesis, such as *Escherichia-Shigella* and *Klebsiella*, are part of healthy commensal microbiota in Ghanaian individuals. A limitation of this study is that it involved microbial profiling based on 16S rRNA sequencing, which is not powerful enough to estimate disease pathogenesis. More detailed species-level analysis is required to discuss the possibility that certain commensal bacteria affect the severity of diarrhea caused by other enteric pathogens. Furthermore, a longitudinal analysis of the intestinal microbiota from the onset of the disease to recovery after therapeutic intervention would have provided important insights into the pathogenesis of diarrhea.

## Methods

### Study population and sample collection

We analyzed stool samples obtained from 80 patients (49 adults, > 19 years old), 13 adolescents (10–19 years old), and 18 children (< 10 years old) attending the Ga West Municipal Hospital, Greater Accra Region, Ghana with diarrhea symptoms. Thirty-four healthy individuals resident in the Eastern Region of Ghana were recruited as controls. Healthy cohort individuals who had been administered antibiotics within 4 weeks prior to sample collection were excluded. All samples were transported to NMIMR and processed for storage within 24 h of sample collection. Stool samples were stored at − 80 °C until DNA extraction.

### Ethical approval

This study was approved by the Institutional Review Board of Noguchi Memorial Institute for Medical Research (NMIMR) (approval number: 096/16-1; dated on May 3, 2017). We confirmed that all methods were performed in accordance with the relevant guidelines and regulations. The written informed consent for sample collection and subsequent analysis was provided by all the participants (healthy individuals as well as patients) prior to enrollment. Regarding under the age of 18 years, informed consent was obtained from a parent and/or legal guardian.

### Preparation of bacterial fractions from fecal samples

Bacterial pellets were prepared from frozen fecal samples. Briefly, 0.5 mL of watery stool was added to the same volume of SM-plus buffer (100 mM NaCl, 50 mM Tris–HCl [pH 7.4], 8 mM MgSO_4_-7H_2_O, 5 mM CaCl_2_-2H_2_O and 0.01% [w/v] gelatin). Bacterial suspensions were then filtered through a 100-μm cell strainer (Corning, Inc., Corning, NY, USA). The filtered bacterial suspension was used for DNA extraction.

### DNA extraction, amplification, and 16S rRNA gene sequencing

DNA was extracted from the fecal sample-derived bacterial fraction as previously described^39^. The 16S rRNA gene libraries were prepared according to the 16S Metagenomics Sequencing Library Preparation guide (Illumina, San Diego, CA, USA, Part #15044223 Rev. B). Briefly, the hypervariable V3-V4 regions of the 16S rRNA gene were amplified using specific primers: forward (5′-ACACGACGCTCTTCCGATCTCCTACGGGNGGCWGCAG-3′) and reverse (5′-GACGTGTGCTCTTCCGATCTGACTACHVGGGTATCTAATCC-3′), including Illumina adapter overhang nucleotide sequences (indicated by underlines)^40^. Next, adapter ligation for PCR amplicons was performed using NEB Next Multiplex Oligos for Illumina (Dual Index Primers Set 1) (New England Biolabs, Ipswich, MA, USA). Sequencing was performed on the Illumina MiSeq (Illumina) using the MiSeq Reagent Kit v3 (600-cycle) with a 20% PhiX (Illumina) spike-in at NMIMR.

### Sequence and statistical analyses

Sequences were quality filtered, denoised, and analyzed with Quantitative Insights Into Microbial Ecology 2 (QIIME 2 version 2019.4)^41^. Briefly, paired-end reads were denoised into amplicon sequence variants with DADA2^42^. Taxonomy was assigned to the resulting amplicon sequence variants against the SILVA database (release 132)^43^, trimmed to the V3-V4 region of the 16S rRNA gene, using the Naive Bayesian classifier^44^. Alpha-diversity measurements and weighted UniFrac distances were calculated using QIIME2 version 2019.4. Data were preprocessed as described in ANCOM-II to remove low-abundance or rare taxa prior to differential abundance analysis^45^. Statistical analysis of metagenomic profiles was performed using STAMP, version 2.0^15^.

2 groups and multigroup analysis were performed by Mann–Whitney test or Kruskal–Wallis *H* test using STAMP. Statistically significant characteristics were further examined by post hoc tests (Tukey–Kramer) to determine which groups of profiles differed from each other. Unassigned reads were analyzed without deleting them. Predicted metabolic pathways and functions were analyzed using PICRUSt software^46^ with the Kyoto Encyclopedia of Genes and Genomes (KEGG) database^47^. Differentially abundant taxa were identified using linear discriminant analysis (LDA) effect size (LEfSe) methods^16^.

### Detection of norovirus and rotavirus from fecal samples

Norovirus single-stranded RNA was extracted directly from the stool using Trizol reagent (Invitrogen, Carlsbad, CA, USA) according to the manufacturer’s instructions and stored at − 70 °C. RT-PCR was performed in a one-step method using the SuperScript III One-Step RT-PCR System with Platinum Taq DNA Polymerase (Invitrogen). Primers based on the 3′ end conserved genome region of the polymerase open reading frame were as follows: GIFFN (F) (sense, 5′-GGAGATCGCAATCTCCTGCCC-3′), GISKR (R) (sense, 5′-CCAACCCARCCATTRTACA-3′). PCR conditions were as follows: 94 °C for 2 min, 40 cycles of PCR with denaturation at 94 °C for 20 s, annealing at 50 °C for 30 s, and extension at 72 °C for 30 s, and an optical read step at 72 °C for 1 min. PCR bands were detected by gel electrophoresis with ethidium bromide. Rotavirus was detected in the stool using the ProSpecT Rotavirus Microplate Assay (Thermo Fisher Scientific, Waltham MA, USA) according to the manufacturer’s instructions.

## Supplementary Information

Supplementary Figure 1.

Supplementary Figure 2.

Supplementary Figure 3.

Supplementary Captions.
