# Supplementary figures and images for: Gut microbiota signature of pathogen-dependent dysbiosis in viral gastroenteritis
Source: Sci Rep. 2021 Jul 6;11:13945. doi: 10.1038/s41598-021-93345-y (PMC8260788; doi:10.1038/s41598-021-93345-y)

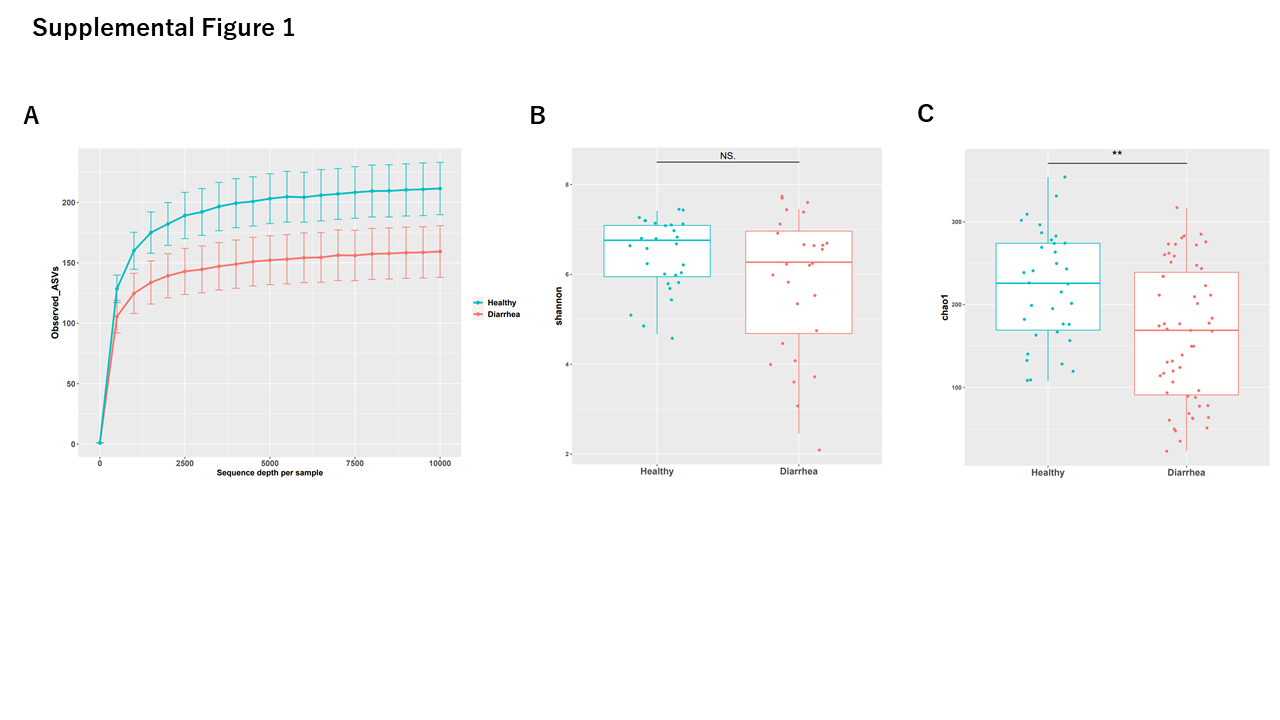

Supplement: Supplementary file 1 — Supplementary Figure 1. [file 41598_2021_93345_MOESM1_ESM.tif]

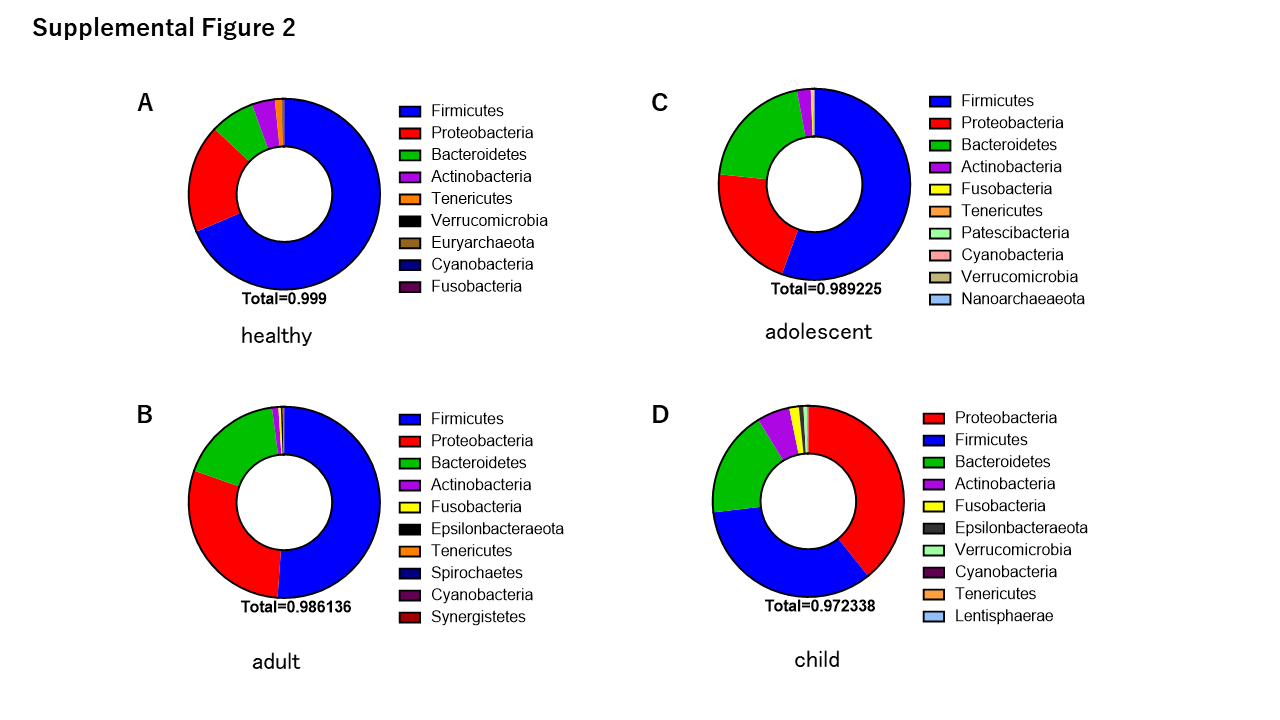

Supplement: Supplementary file 2 — Supplementary Figure 2. [file 41598_2021_93345_MOESM2_ESM.tif]

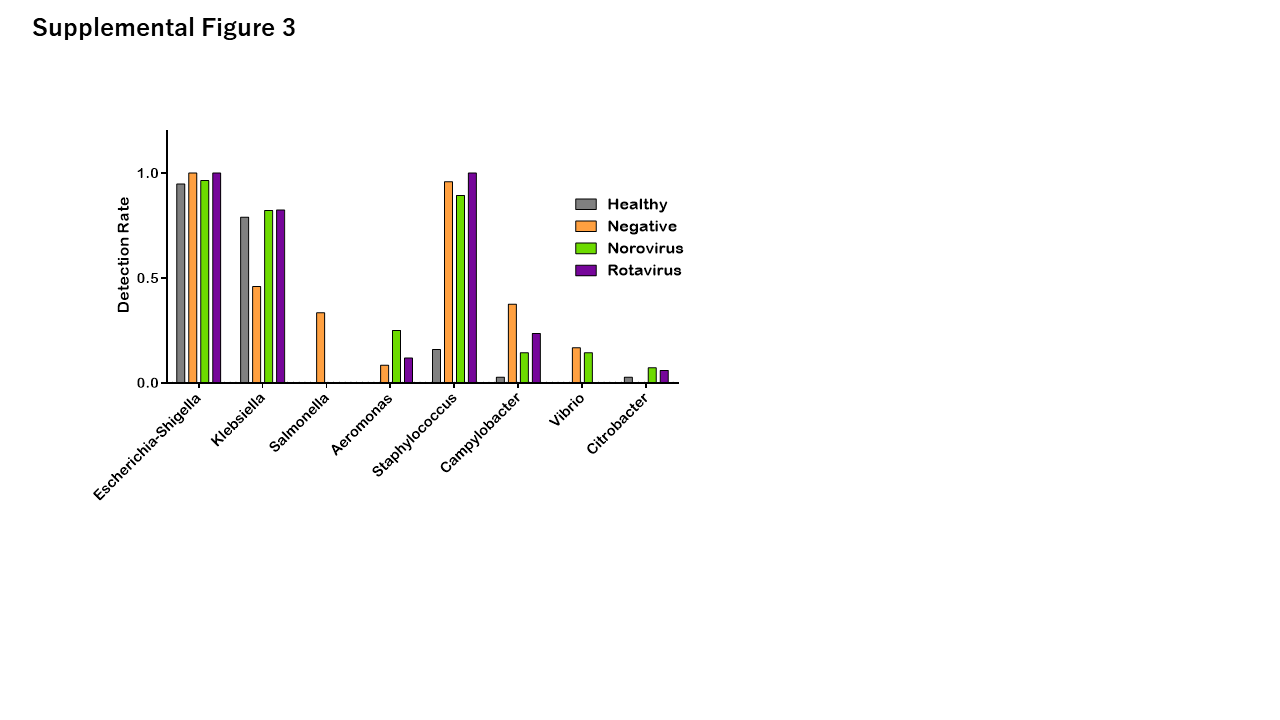

Supplement: Supplementary file 3 — Supplementary Figure 3. [file 41598_2021_93345_MOESM3_ESM.tif]
