# Supplementary material for: Gut microbiota signature of pathogen-dependent dysbiosis in viral gastroenteritis
Source: Sci Rep. 2021 Jul 6;11:13945. doi: 10.1038/s41598-021-93345-y (PMC8260788; doi:10.1038/s41598-021-93345-y)
Supplement: Supplementary file 4 — Supplementary Captions. [file 41598_2021_93345_MOESM4_ESM.docx]

**Supplemental** **Figure 1. Comparison of fecal microbiota between adult patients with diarrhea and healthy adult cohorts**

(**A**) Rarefaction curves showing the number of observed amplicon sequence variants between individuals with diarrhea and healthy controls. Error bars indicate confidence intervals. Alpha diversity (Shannon) (**B**) is fairly lower in individuals with diarrhea, compared with healthy controls. Though not significant. Gut bacteria richness (Chao1) (**C**) is significantly (q = 0.002) reduced in individuals with diarrhea, compared with healthy controls. ** indicate significant differences with Kruskal–Wallis test, q < 0.005.

**Supplemental** **Figure 2. Taxonomic profiles (phylum) of fecal bacteria in patients with diarrhea and healthy controls**

Top 10 relative abundance of fecal taxa at the phylum level in healthy adult controls (A) and patients with diarrhea; adults (B), adolescents (C), and children (D).

**Supplemental Figure 3. Co-infection of bacterial pathogen in enterovirus negative or positive diarrheal patients**

Bar graph showing detection rates of microbes in norovirus, rotavirus or none-virus positive patients with diarrhea.
